# Professionalism in traditional Chinese medicine (TCM) practitioners: a qualitative study

**DOI:** 10.1186/s12906-020-03127-8

**Published:** 2020-11-09

**Authors:** Yu Heng Kwan, Sarah Chooi, Sungwon Yoon, Xiang Ling Ang, Jie Kie Phang, Hwee Ling Koh, Julian Thumboo, Swee Cheng Ng, Warren Fong

**Affiliations:** 1 Program in Health Services and Systems Research, Duke-NUS Medical School, Singapore, Singapore; 2 Department of Pharmacy, National University of Singapore, Singapore, Singapore; 3 Singapore Thong Chai Medical Institution, Singapore, Singapore; 4 Department of Rheumatology and Immunology, Singapore General Hospital, Singapore, Singapore; 5 NUS Yong Loo Lin School of Medicine, National University of Singapore, Singapore, Singapore; 6 Duke-NUS Medical School, Singapore, Singapore

**Keywords:** Professionalism, Traditional Chinese medicine, TCM practitioners, Singapore, Qualitative, Assessment

## Abstract

**Background:**

Medical professionalism is important for establishing public trust in doctors. Its definition is culture-sensitive. However, no research has explored medical professionalism in Traditional Chinese Medicine (TCM) practitioners. This study aims to (a) establish the domains of professionalism in TCM practitioners in Singapore, and to (b) compare the domains of professionalism of practitioners trained in TCM and practitioners trained in conventional medicine.

**Methods:**

Data for this qualitative study was collected through in-depth interviews (IDIs) with TCM practitioners. IDIs were audio-recorded and transcribed verbatim. Thematic analysis was conducted by two independent coders using the Professionalism Mini-Evaluation Exercise (P-MEX) as a framework. The domains of professionalism in TCM and conventional medicine were then compared, using data from a similar study on professionalism in practitioners trained in conventional medicine.

**Results:**

A total of 27 TCM practitioners (40.7% male) participated in this study. The four domains of professionalism in the P-MEX, namely doctor-patient relationship skills, reflective skills, time management and inter-professional relationship skills, and two new sub-domains, “communicated effectively with patient” and “demonstrated understanding and integrated with conventional medicine”, were relevant to TCM practitioners. This is largely similar to that of practitioners trained in conventional medicine, with a few differences, including “ensured continuity of care” and “used health resources appropriately”.

**Conclusion:**

The domains of professionalism in TCM practitioners were established and they are similar to that of practitioners trained in conventional medicine. This study is the first to define medical professionalism in TCM practitioners. Findings will provide guidance on the education of professionalism in TCM practitioners.

**Supplementary Information:**

The online version contains supplementary material available at 10.1186/s12906-020-03127-8.

## Background

Medical professionalism in healthcare is defined as a set of values, behaviours and relationships that underpins public trust in doctors [1]. Professional conduct is associated with increased patient trust, satisfaction and compliance to treatment, which leads to improved medical outcomes [2]. On the other hand, unprofessional conduct in doctors is linked to an increased risk of adverse medical outcomes in patients [3]. To ensure better medical outcomes for patients, all doctors in healthcare, not just practitioners trained in conventional medicine but also Traditional Chinese Medicine (TCM) practitioners, should cultivate good medical professionalism.

In recent decades, changes in the healthcare delivery systems have caused the public to question the professionalism of doctors [4, 5]. With access to web-based information, patients are more informed, and may have higher expectations of doctors in terms of medical knowledge and communication as compared to the past [6]. As a result, there has been growing emphasis on the teaching of professionalism in undergraduate and post-graduate medical schools [4, 7]. Concurrently, there is a corresponding increase in medical literature attempting to give “professionalism” a clearer definition [4, 8]. The Physicians Charter on Medical Professionalism which defines a list of principles and professional responsibilities [5] has been endorsed by 108 national and international organisations to date [9]. The charter defines professionalism as a commitment to professional competence and responsibilities, upholding honesty with patients, maintaining patient confidentiality, maintaining appropriate patient-practitioner relationships, improving quality of care, ensuring just distribution of limited resources, having scientific knowledge and managing conflicts of interest [5]. Extensive research on medical professionalism, mainly in Western countries [8], has attempted to define professionalism through qualitative methods [10, 11]. For example, Jha et al. defined themes of professionalism as compliance to values, patient access, doctor-patient relationship, professional management, demeanour, personal awareness and motivation [10]. In another study, Wagner et al. further defined knowledge/technical skills, character values and patient relationship as core themes of professionalism [11].

However, research has also shown that professionalism can be influenced by culture [12, 13]. Studies on professionalism in conventional medicine in the Chinese cultural context, in particular, have shown that views of professionalism are influenced by China’s long-established Confucian and cultural traditions [13–15]. For example, the Chinese Medical Doctor Declaration, which is the Physicians Charter adapted to the Chinese context, defines professionalism as such: equality and benevolence, primacy of patients, honesty and fidelity to promises, commitment to excellence and prudence, incorruptibility and impartiality, and lifelong learning [13]. Therefore, any definitions of professionalism need to be validated with respect to the culture and context it is applied to [8].

TCM practitioners are clinicians who practise TCM, which is a long-established medicinal practice rooted in traditional Chinese values, and consists of therapies such as herbal medicine and acupuncture which are primarily based on traditional theories [16, 17]. TCM is an important area of study because it is practised in many parts of the world, is recognised as a profession in some countries [18], and interest in TCM is growing worldwide [19]. In recent years, clinical internship and training, at which TCM students are expected to learn and model after the professionalism of their TCM practitioner preceptors, has become an important part of TCM education [20, 21]. The focus on professionalism in TCM is probably also in response to the change in patients’ expectations of doctors as they become more informed [6]. This is seen in research in Hong Kong which has shown that more educated patients, who tend to be younger, more westernised and have less attachment to Chinese cultural values, may have less trust in TCM practitioners compared to patients with lower education levels [22]. Therefore, there is still room for improvement of public trust in TCM practitioners [23].

Despite the knowledge that professionalism is culture-sensitive, no studies so far have attempted to define professionalism in Traditional Chinese Medicine (TCM) practitioners. Therefore, there is a need to define professionalism in TCM practitioners, in the form of professional traits and behaviours. This is because it is best to assess professionalism in TCM students by the “does” in the Miller’s pyramid, which is the student’s performance in the work environment [24], using assessment tools such as the Professionalism Mini-Evaluation Exercise (P-MEX). This can guide the education and assessment of professionalism in future TCM practitioners, and potentially help to increase public trust in the profession. To study professionalism in TCM, Singapore provides a unique setting. Singapore is one of the few countries which formally recognises the role of TCM in its healthcare system, with legal regulations on TCM practice implemented through the Traditional Chinese Medicine Practitioners Act [25, 26]. Registration of TCM practitioners under the Act commenced in year 2000 [27], and to date 3004 TCM practitioners have registered [28]. Singapore’s healthcare system is based on its established conventional medical system, and TCM is recognised as a complementary healthcare system that is structurally separate from the conventional medical system [29]. Even though it is a complementary medical system, TCM is commonly used among Singapore’s population [30], motivated by deep-seated cultural beliefs as well as patients’ own initiative to seek supportive treatment to conventional Western therapies [27]. Furthermore, Singapore has been recognised internationally for its progress in TCM and good policies in the regulation of TCM [31].

In this study, we aim to: (a) establish the domains of professionalism of TCM practitioners and (b) compare the domains of professionalism of practitioners trained in TCM and practitioners trained in conventional medicine.

## Methods

### Study design

We used in-depth interviews (IDIs) to elicit TCM practitioners’ views of medical professionalism in TCM, from 1st July 2019 to 31st August 2019. We chose IDIs as the method of data collection because it can elicit in-depth information about participants’ personal views and experiences [32]. The Professionalism Mini-Evaluation Exercise (P-MEX) [33] was used as a guide to design the standardised, semi-structured interview guide (supplementary material). The P-MEX is a tool used to assess professionalism in medical students and residents, and consists of 4 main domains: doctor-patient relationship skills, reflective skills, time management and inter-professional relationship skills [33]. It is used as the basis for the interview guide as it is shown to have strong evidence as a reliable medical professionalism evaluation tool [34]. The interview guide was pilot tested. Each IDI with a TCM practitioner was conducted in English or Mandarin, by one of the two trained facilitators who are fluent in both English and Mandarin. Each IDI lasted approximately 30–60 min and was divided into two sections. Firstly, TCM practitioners were asked to describe and elaborate on characteristics of professionalism that they consider important for a TCM practitioner. Next, TCM practitioners were asked if there were any characteristics listed in the P-MEX that they think are not relevant, and to choose up to five least relevant characteristics. In addition, TCM practitioners were also invited to list any missing items which may be important for the assessment of a professional TCM practitioner. After the interview, TCM practitioners were asked to complete a brief demographic questionnaire. IDIs were conducted until data saturation occurred.

We anchored the methodology with reference to the Consolidated Criteria for Reporting Qualitative Research (COREQ) checklist [35].

### Participants

TCM practitioners practising in a range of TCM clinics and institutions in Singapore were invited to participate in the study. A purposive sample, based on age, gender, duration of practice and nature of practice setting, was identified. In Singapore, TCM practitioners practise in either voluntary organisations which are non-profit TCM organisations that run on charity donations to provide low cost TCM treatment to patients, or for-profit private organisations which provide relatively more costly TCM treatments. We included practising TCM practitioners who are registered with the Singapore TCM Practitioners Board as a TCM practitioner, and have at least 2 years of experience in TCM practice at the time of data collection.

### Data analysis

All IDIs were audio-recorded and transcribed verbatim. Interviews conducted in Mandarin were translated and transcribed in English. Thematic data analysis was undertaken by two independent coders (SC and JKP), both trained in qualitative research. A hybrid approach of thematic analysis was employed, which included both inductive approach based on the grounded theory and deductive approach using a template of codes [36]. The four domains of medical professionalism from the P-MEX were used as a coding template in the NVivo 11 software for data analysis. Any disagreements regarding the coding were discussed among the research team until a consensus was reached. Overall, the relevance/lack of relevance of sub-domains were determined by vote count in the second part of the interview when the TCM practitioners were asked to choose up to five least relevant items in assessing professionalism.

We combined the data from our previous study on professionalism in practitioners trained in conventional medicine in Singapore, which also used P-MEX as the coding framework [37]. We then compared the domains of professionalism derived from TCM practitioners, with the domains of professionalism derived from practitioners trained in conventional medicine.

### Ethics

This study is approved by the SingHealth Centralised Institutional Review Board (Ref No. 2016/3009). Informed written consent was obtained from all participants before every interview. The study was conducted in alignment with the principles of the 1964 Declaration of Helsinki.

## Results

A total of 27 TCM practitioners [40.7% male, median age (range) 33 years old (27 to 77 years)] participated in the IDIs (14 conducted in English and 13 conducted in Chinese). The median (range) number of years of experience of the TCM practitioners was 8 years (2 to 44 years). 48.1% of the TCM practitioners practised in a voluntary organisation setting, while 51.9% of them practised in a for-profit setting. Data saturation occurred after 24 IDIs, with no new themes emerging. The general and individual socio-demographic characteristics of the TCM practitioners who participated in the IDIs are summarised in Table 1 and Supplementary Table 1 respectively.

**Table 1:**
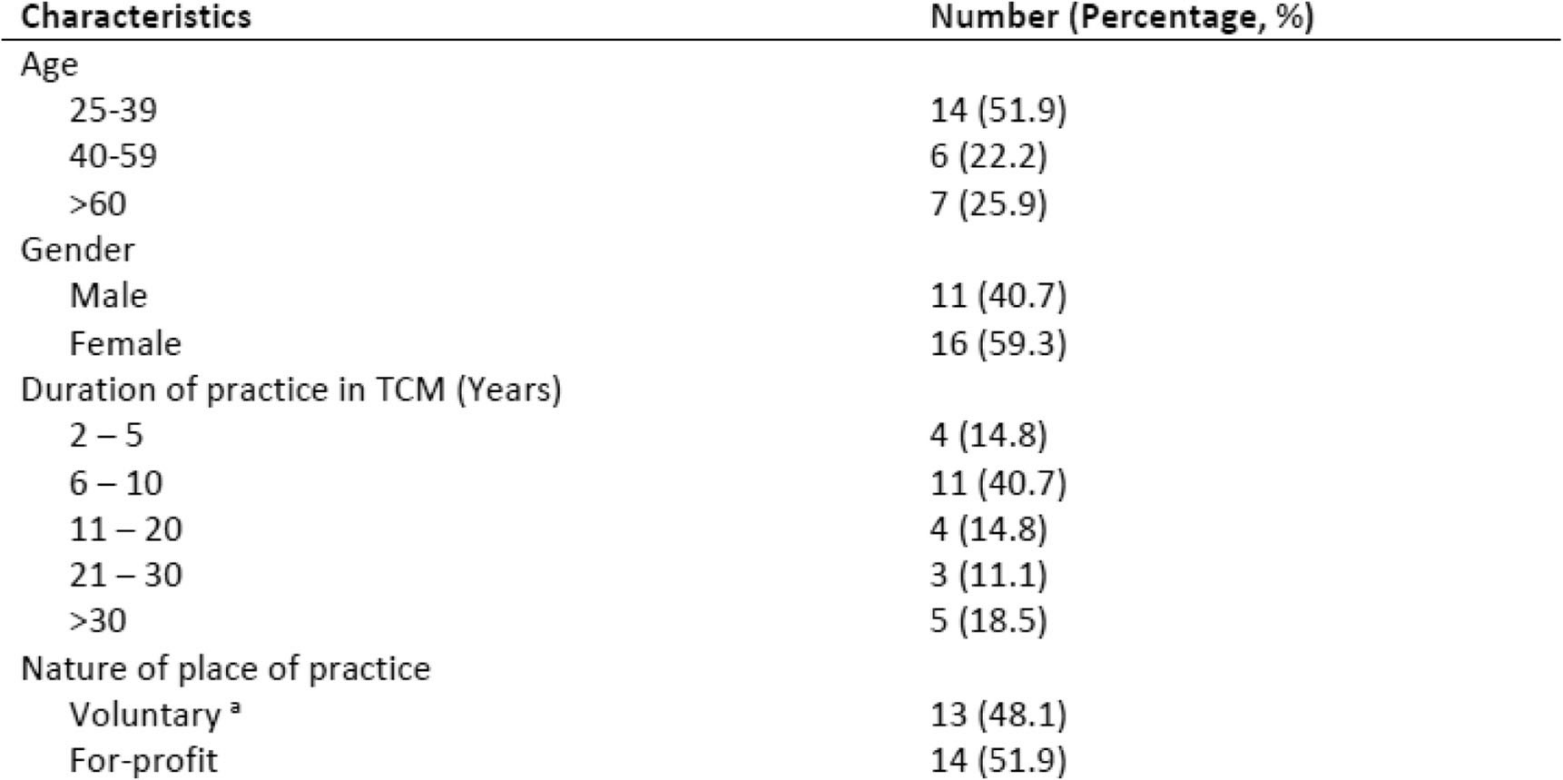
Demographic profile of TCM practitioners who participated in interviews (*n* = 27)

^a^refers to charitable TCM clinics

### Framework of medical professionalism

**Fig. 1:**
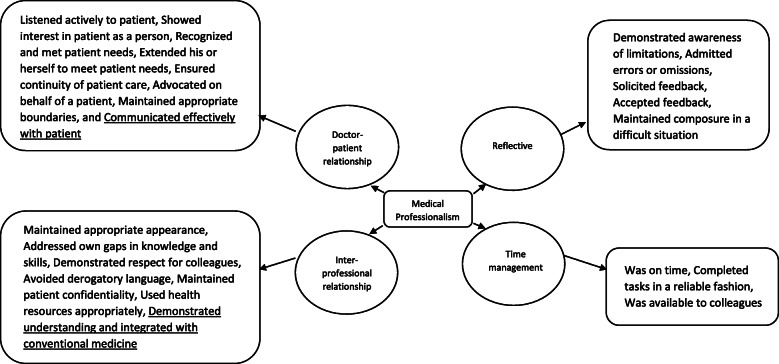
Domains and subdomains of medical professionalism for TCM practitioners. Underlined sub-domains are the new sub-domains derived from this study

Using the P-MEX as an a priori framework for medical professionalism, 4 domains (doctor-patient relationship skills, reflective skills, time management and inter-professional relationship skills) were elicited (Fig. 1). Out of the 21 sub-domains in the P-MEX, we identified 17 sub-domains relevant to TCM practitioners, and 4 sub-domains considered less relevant. In addition, 2 new sub-domains were derived from this study. The results are shown in Table 2.

**Table 2:**
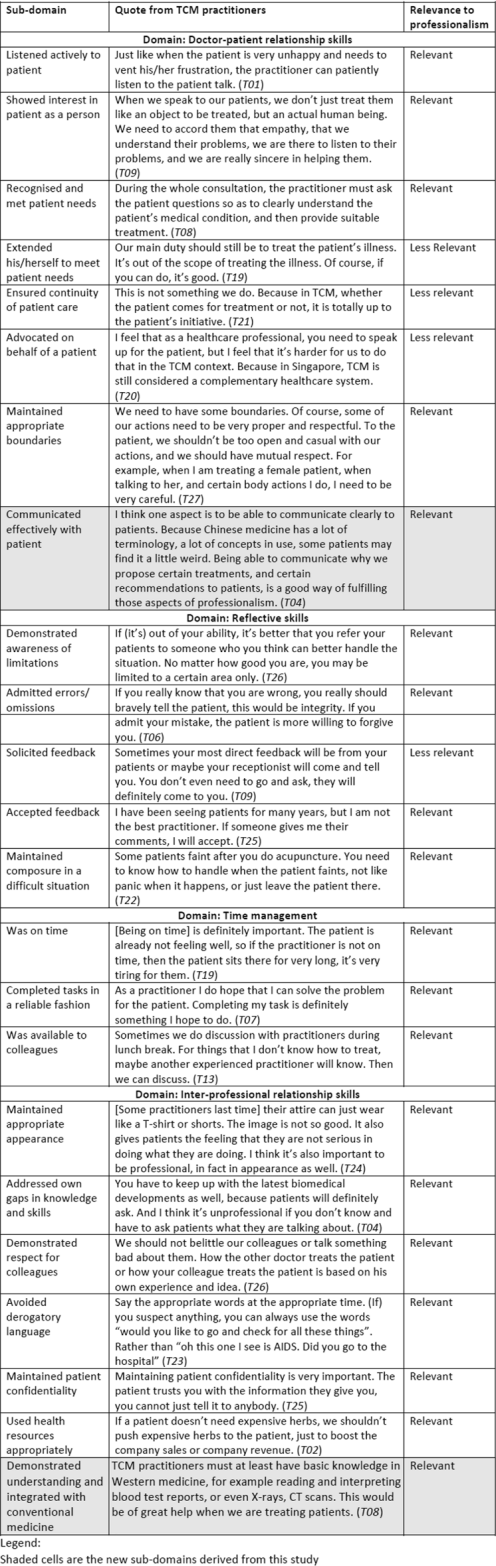
Representative quotes from interviews with TCM practitioners and their implications on the relevance of each P-MEX domain to professionalism

Shaded cells are the new sub-domains derived from this study

### Doctor-patient relationship skills

Our data corresponded to all 7 original sub-domains under doctor-patient relationship skills in the P-MEX. They are (1) listened actively to patient, (2) showed interest in patient as a person, (3) recognised and met patient needs, (4) extended his/herself to meet patient needs, (5) ensured continuity of care, (6) advocated on behalf of a patient, and (7) maintained appropriate boundaries.

In addition, a new sub-domain “communicated effectively with patient” was derived from this study. TCM practitioners highlighted the importance of explaining the patient’s condition, the treatments and recommendations to the patients clearly, in a way that the patient can understand. This is especially so in the TCM context, which involves many complex Chinese terms and concepts that practitioners need to be able to explain in a comprehensible way.

“The TCM practitioner should be clear on how the patient’s health condition is, sometimes we (as practitioners) need to explain to the patient what body constitution they belong to, what kind of medications they are eating now, how should their medical condition be treated. Sometimes when there are some patients who do not have a clear understanding, then it is very difficult for them to adhere to treatment.” (T01)

TCM practitioners deemed the sub-domains “extended his/herself to meet patient needs”, “ensured continuity of patient care” and “advocated on behalf of a patient” to be less relevant. On extending extra effort to help patients, practitioners felt that although it is good to make an additional effort to support patients, it is not necessary to do beyond the scope of their medical duties. TCM practitioners also felt that they have limited capacity to ensure the proper transition of patient care because in the TCM setting, it is dependent on the patient’s initiative to go for the recommended treatment. In terms of advocating for the patient, TCM practitioners highlighted that as TCM is a complementary health system in Singapore’s context, they find it difficult to speak up for the patient.

On the reason why “extended his/herself to meet patient needs” is less relevant:

“There may be patients who after getting very close to you … They may ask you for a lot of things they may be over-dependent on you. Like getting certain things done for their family members, maybe even applying for certain subsidy, getting certain application forms done. These are not supposed to be the practitioner’s job.” (T02)

On the reason why “ensured continuity of patient care” is less relevant:

“If the patient is being discharged from the hospital, then the handover of care is important. But in our TCM context, we don’t have such hospital stays, we all work in the clinic setting.” (T06)

On the reason why “advocated on behalf of a patient” is less relevant:

“In TCM line, because we cannot really apply for subsidies and others for the patient, so I don’t think it’s really applicable for us.” (T14)

### Reflective skills

Our data corresponded to the 5 original sub-domains under reflective skills, namely, (1) demonstrated awareness of limitations, (2) admitted errors/omissions, (3) solicited feedback, (4) accepted feedback, and (5) maintained composure in a difficult situation.

The sub-domain “solicited feedback” was deemed to be less relevant by TCM practitioners. TCM practitioners felt that important feedback will be given to them by patients or colleagues, even without actively asking for it.

### Time management

All 3 original sub-domains under time management were found to be relevant in our data, namely, (1) was on time, (2) completed tasks in a reliable fashion, and (3) was available to colleagues.

Many TCM practitioners highlighted that while they should always strive to complete consultations on time, there are times where a consultation may inevitably take longer than expected. For example, first-time patients or complicated cases which require a longer time to manage, under which the TCM practitioner would prioritise meeting the patient’s need over completing the task within time limits.

“If the practitioner is late because the previous patient needs special attention, and the practitioner needs to give more time to that patient, I think this is understandable, and I will not consider this as unprofessional.” (T07)

### Inter-professional relationship skills

Under inter-professional relationship skills, all 6 original sub-domains were found to be relevant in our data. They are (1) maintained appropriate appearance, (2) addressed own gaps in knowledge and skills, (3) demonstrated respect for colleagues, (4) avoided derogatory language, (5) maintained patient confidentiality and (6) used health resources appropriately.

In addition, “demonstrated understanding and integrated with conventional medicine” was a new sub-domain which emerged in this study. TCM practitioners highlighted that it is necessary to at least have basic knowledge in conventional medicine, and be able to read and interpret lab reports, X-rays and CT scan reports. This would help give them a better and more comprehensive understanding of the patient’s condition and identify whether and how TCM can complement conventional medical treatment.

“If we cannot diagnose the patient’s illness using TCM theory and methods, then we will tell the patient to go for a blood test and to let me see the report after that. Or we tell them to go to the Western doctor to check their liver, to check their womb, to see if there is any issue. And then bring the Western medical report results to let us see. Then we use TCM methods to help the patient.” (T11)

Some TCM practitioners also expressed hope for better integration of care and communication between practitioners trained in conventional medicine and TCM practitioners in the future.

“The western doctors thought TCM practitioners just take the pulse then prescribe medicine. They didn’t know that TCM practitioners also know scientific knowledge. They thought the TCM practitioners don’t know estrogen or progesterone.” (T05)

### Least relevant sub-domains as deemed by TCM practitioners

The least relevant sub-domains selected by the TCM practitioners were “advocated on behalf of the patient” (*n* = 24), “extended his/herself to meet patient needs” (*n* = 16), “solicited feedback” (*n* = 16), and “ensured continuity of patient care” (*n* = 14).

### Comparison of professionalism between TCM practitioners and practitioners trained in conventional medicine

Comparing the domains of professionalism in TCM practitioners against the domains of professionalism in practitioners trained in conventional medicine [37], there was a broad similarity with a few key differences, as reflected in Table 3. Notably, the new sub-domain which emerged from both studies, “communicated effectively with patient”, was relevant to professionalism in both TCM practitioners and practitioners trained in conventional medicine.

**Table 3:**
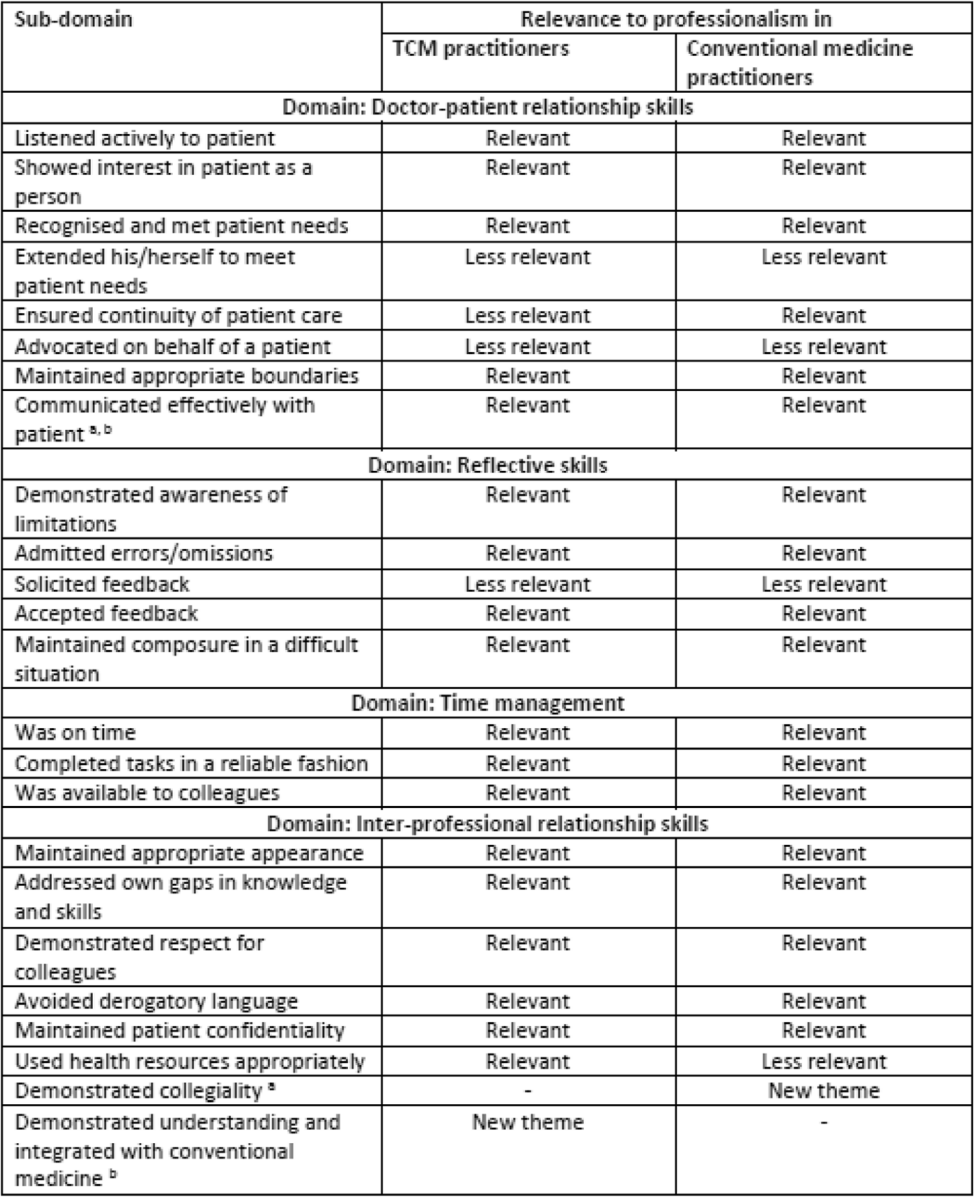
Comparison of relevance of each P-MEX domain to professionalism between TCM practitioners and conventional medicine practitioners

^a^new sub-domain derived from the study in conventional medicine practitioners

^b^new sub-domain derived from the study in TCM practitioners

The key differences in professionalism between TCM practitioners and practitioners trained in conventional medicine are as follows: the sub-domain “ensured continuity of patient care” was deemed to be relevant to professionalism in practitioners trained in conventional medicine, but it was deemed less relevant to professionalism in TCM practitioners. Additionally, the sub-domain “used health resources appropriately” was deemed relevant to professionalism in TCM practitioners, but it was less relevant to professionalism in practitioners trained in conventional medicine.

## Discussion

This qualitative study is the first study to establish the domains of professionalism in TCM practitioners, and to compare this with practitioners trained in conventional medicine [37]. The ‘Ethical Code and Ethical Guidelines for TCM Practitioners’ from the Singapore TCM Practitioners Board represents the essential doctrines of conduct of TCM practitioners [38]. The ethical guidelines cover the following main areas- (1) Standard of Good TCM Practice, (2) Relationships with Patients, (3) Information about TCM Practitioners’ Services, (4) TCM Practitioners in a Non-Medical Context, (5) Financial and Commercial Conflicts of Interest, (6) Issues of Fitness to Practise. Except the ‘Information about TCM Practitioners’ Services’ and ‘Issues of Fitness to Practise’ which are not usually considered as part of professionalism, the ethical guidelines from the Singapore TCM Practitioners Board can be largely mapped to the domains and subdomains in the P-MEX.

In establishing the domains of professionalism in TCM practitioners, our study identified new sub-domains, which are not present in the original P-MEX, for assessment of professionalism in TCM practitioners. These are “communicated effectively with patient” under the doctor-patient relationship skills domain, and “demonstrated understanding and integrated with conventional medicine” under the inter-professional relationship skills domain. The new sub-domain “communicated effectively with patient” reflects the increasing emphasis on patient autonomy in healthcare [39]. Modern technology allows patients greater access to information on health issues. Therefore, patients expect more time to be spent on patient-practitioner communication, to go through information that they might have read, and also to explain their condition and tests in greater detail [6]. The emergence of “demonstrated understanding and integrated with conventional medicine” shows a shift to the integration of TCM in healthcare, where TCM and conventional medicine complement each other to bring greater benefits to patients [17]. An institutional separation of conventional and TCM medical systems has been a barrier to allowing patients to fully discuss their concerns about the concurrent use of both TCM and conventional therapies with conventional medicine practitioners and TCM practitioners [26, 30]. Therefore, better mutual understanding of both TCM and conventional medicine should be encouraged for potential safer, faster and more effective patient care [40].

When comparing the domains of professionalism in TCM practitioners against that of practitioners trained in conventional medicine, this study found two key differences. One of the key differences is in the sub-domain “ensured continuity of patient care”, which was less relevant to professionalism for TCM practitioners, but was relevant to practitioners trained in conventional medicine. This may be because TCM practitioners do not have to assume the responsibility for care continuity for the patient. While TCM practitioners are an important part of the care team, they do not usually coordinate care for patients, and do not have to accept overall responsibility for patient care. The care coordination is a role traditionally assumed by the clinicians from the conventional medicine [41]. Another reason may be due to the outpatient clinic setting of TCM, along with the lack of a formal inter-professional referral system between TCM and other healthcare professions [27], which limits the proper handover of care from TCM practitioners to other healthcare professionals. Many TCM practitioners highlighted that they usually advised patients to seek further consultation with other health professionals, but would not make a formal referral for patients. Compared to the hospital setting where the practitioners trained in conventional medicine interviewed worked, continuity of care in the TCM context is poor. Similar situations have also been encountered in other countries, such as Hong Kong, where there is also no formal inter-professional referral system between TCM practitioners and other healthcare professionals. Under such circumstances, continuity of care is highly dependent on patients’ initiative to seek appropriate medical care at the advice of their TCM practitioners [42].

The second key difference is in the sub-domain “used health resources appropriately”, which was relevant to professionalism for TCM practitioners but was less relevant to practitioners trained in conventional medicine. This is possibly because of the holistic treatment approach in TCM, where treatment is tailored to the individual based on the TCM concept of syndrome differentiation [17, 43]. The personalised treatment approach in TCM also leads to some difficulty in forming guideline recommendations for TCM treatments [44] and for TCM practitioners to follow TCM treatment guidelines fully [45]. This is in contrast to disease-targeted practice in conventional medicine, where there is a strict treatment guideline for each disease [43]. Therefore, TCM practitioners likely have more responsibility and control of health resources used on every patient, based on their own professional judgement. Whereas in conventional medicine, the use of health resources may be more strictly controlled by clinical practice guidelines which dictate the standard therapy protocols, hence practitioners trained in conventional medicine may have less control over health resources. Coupled with the lack of coverage of TCM treatments under the MediSave scheme (Singapore’s national medical savings scheme) [46], the judicious use of health resources by TCM practitioners may thus be deemed to be more important.

The strengths of this study include purposive sampling of the TCM practitioners in Singapore. The sample’s gender profile and distribution of practice setting mirrors that of currently practising TCM practitioners in Singapore [28]. In addition, we ensured the inclusion of a wide range of ages and years of experience. Therefore, our data captured a broad range of views and is representative of the views of TCM practitioners in Singapore on professionalism.

The limitations of this study include the limited generalisability of the findings to other countries as professionalism is often affected by different cultural and social contexts. However, being the first study to explore medical professionalism in TCM practitioners, this study can set the stage for future research on professionalism in TCM practitioners in other countries. Although the sample gender distribution (40.7% male) mirrors the gender distribution of the currently practising TCM practitioner population in Singapore (45.3% male) [28], the distribution of practice setting in our study (48.1% in voluntary setting) may be slightly different from the practicing TCM practitioner population in Singapore (31.1% in voluntary setting). However, no distinct differences were observed for practitioners from different practice settings. Findings from this study may not be wholly applicable to junior TCM practitioners with little clinical experience as we included only experienced TCM practitioners. Recognising that professionalism is influenced by clinical experience [47], further study is warranted to explore professionalism among junior TCM practitioners. This can allow comparison of the domains/subdomains of professionalism between junior and experienced TCM practitioners, as well as examination of whether the new sub-domains identified in this study are the result of clinical experience. In addition, while the aim of this study was to establish domains of professionalism in TCM practitioners, the exclusive use of P-MEX as a guiding framework for the interview guide and data analysis could have left limited space to explore other unexpected aspects of the TCM practitioners’ medical professionalism. Hence, caution is warranted when interpreting the findings. A more reflective and critical understanding of TCM practitioners’ professional behaviors requires further exploration.

## Conclusion

In conclusion, doctor-patient relationship skills, reflective skills, time management and inter-professional relationship skills are domains of professionalism in TCM practitioners. Our study further identified two new sub-domains “communicated effectively with patient” and “demonstrated understanding and integrated with conventional medicine” that were important in the assessment of professionalism in TCM practitioners. The domains of professionalism in TCM practitioners were largely similar to that of practitioners trained in conventional medicine, except in the sub-domains of “ensured continuity of care” and “used health resources appropriately”, which could be attributed to structural differences between the TCM and conventional medicine healthcare systems. This study provided an understanding of professionalism in TCM practitioners, which can be useful for the education and assessment of professionalism in current and future TCM practitioners.

## Supplementary Information

**Additional file 1.**

**Additional file 2.**

## Data Availability

The datasets used and/or analysed during the current study are available from the corresponding author on reasonable request.

## Abbreviations

COREQ: Consolidated Criteria for Reporting Qualitative Research
FGD: Focus group discussion
IDI: In-depth interviews
P-MEX: Professionalism Mini Evaluation Exercise
TCM: Traditional Chinese Medicine

## References

[1] Working Party of the Royal College of Physicians (2005). Doctors in society. Medical professionalism in a changing world. Clin Med (Lond).

[2] Mueller PS (2015). Teaching and assessing professionalism in medical learners and practicing physicians. Rambam Maimonides Med J.

[3] Cooper WO, Spain DA, Guillamondegui O, Kelz RR, Domenico HJ, Hopkins J, Sullivan P, Moore IN, Pichert JW, Catron TF, et al. Association of Coworker Reports about Unprofessional Behavior by surgeons with surgical complications in their patients. JAMA Surg. 2019;9:828–34. 10.1001/jamasurg.2019.1738 PMC6585020 31215973

[4] Cruess SR, Cruess RL (2012). Teaching professionalism - why, what and how. Facts Views Vision ObGyn.

[5] ABIM Foundation. American Board of Internal Medicine. Medical professionalism in the new millennium: a physician charter. Ann Intern Med. 2002;136:243–6. 10.7326/0003-4819-136-3-200202050-00012 11827500

[6] Neo LF (2011). Working toward the best doctor-patient communication. Singap Med J.

[7] Swick HM, Szenas P, Danoff D, Whitcomb ME (1999). Teaching professionalism in undergraduate medical education. JAMA.

[8] Hodges BD, Ginsburg S, Cruess R, Cruess S, Delport R, Hafferty F, Ho MJ, Holmboe E, Holtman M, Ohbu S (2011). Assessment of professionalism: recommendations from the Ottawa 2010 Conference. Med Teach.

[9] Endorsements of The Charter [https://abimfoundation.org/what-we-do/physician-charter/endorsements-of-the-charter].

[10] Jha V, Bekker H, Duffy S, Roberts T (2006). Perceptions of professionalism in medicine: a qualitative study. Med Educ.

[11] Wagner P, Hendrich J, Moseley G, Hudson V (2007). Defining medical professionalism: a qualitative study. Med Educ.

[12] Chandratilake M, McAleer S, Gibson J (2012). Cultural similarities and differences in medical professionalism: a multi-region study. Med Educ.

[13] Jin P (2015). The physician charter on medical professionalism from the Chinese perspective: a comparative analysis. J Med Ethics.

[14] Ho MJ, Yu KH, Hirsh D, Huang TS, Yang PC (2011). Does one size fit all? Building a framework for medical professionalism. Acad Med.

[15] Leung DC, Hsu EK, Hui EC (2012). Perceptions of professional attributes in medicine: a qualitative study in Hong Kong. Hong Kong Med J.

[16] Park HL, Lee HS, Shin BC, Liu JP, Shang Q, Yamashita H, Lim B (2012). Traditional medicine in China, Korea, and Japan: a brief introduction and comparison. Evid Based Complement Alternat Med.

[17] Xu Q, Bauer R, Hendry BM, Fan T-P, Zhao Z, Duez P, Simmonds MSJ, Witt CM, Lu A, Robinson N (2013). The quest for modernisation of traditional Chinese medicine. BMC Complement Altern Med.

[18] WHO Global Report on Traditional and Complementary Medicine 2019 [https://www.who.int/traditional-complementary-integrative-medicine/WhoGlobalReportOnTraditionalAndComplementaryMedicine2019.pdf?ua=1].

[19] TCM is going global as more countries show interest. China Daily [http://www.chinadaily.com.cn/a/201803/03/WS5a9a99c2a3106e7dcc13f5d6.html].

[20] Ji LH (2018). Research on the model of training for skilled professionals in Traditional Chinese Medicine. Technol Econ Guide.

[21] Moore A, Canaway R, O'Brien KA (2010). Chinese medicine Students' preparedness for clinical practice: an Australian survey. J Altern Complement Med.

[22] Rochelle TL, Yim KH (2014). Factors associated with utilisation of traditional Chinese medicine among Hong Kong Chinese. Psychol Health Med.

[23] Chan K, Tsang L (2018). Public attitudes toward traditional Chinese medicine and how they affect medical treatment choices in Hong Kong. Int J Pharm Healthc Mark.

[24] Miller GE (1990). The assessment of clinical skills/competence/performance. Acad Med.

[25] Shimbo M, Nakamura K, Jing Shi H, Kizuki M, Seino K, Inose T, Takano T (2005). Green tea consumption in everyday life and mental health. Public Health Nutr.

[26] Chang L, Basnyat I (2015). Negotiating biomedical and traditional Chinese medicine treatments among elderly Chinese Singaporean women. Qual Health Res.

[27] Luk PLP (2017). Asian pathways of healings: communications in using traditional Chinese medicine in Singapore.

[28] Traditional Chinese Medicine Practitioners Board (2018). Annual Report 2018.

[29] Healthcare Services and Facilities [Internet] [https://www.moh.gov.sg/our-healthcare-system/healthcare-services-and-facilities].

[30] Lim MK, Sadarangani P, Chan HL, Heng JY (2005). Complementary and alternative medicine use in multiracial Singapore. Complement Ther Med.

[31] TCM can play a part in treating Singapore's ageing population: Gan Kim Yong. Channel News Asia [https://www.channelnewsasia.com/news/singapore/tcm-can-play-a-part-in-treating-singapore-s-ageing-population-ga-7746902].

[32] Dicicco-Bloom B, Crabtree BF (2006). The qualitative research interview. Med Educ.

[33] Cruess R, McIlroy JH, Cruess S, Ginsburg S, Steinert Y (2006). The professionalism mini-evaluation exercise: a preliminary investigation. Acad Med.

[34] Kwan YH, Png K, Phang JK, Leung YY, Goh H, Seah Y, Thumboo J, Ng ASC, Fong W, Lie D (2018). A systematic review of the quality and utility of observer-based instruments for assessing medical professionalism. J Grad Med Educ.

[35] Tong A, Sainsbury P, Craig J (2007). Consolidated criteria for reporting qualitative research (COREQ): a 32-item checklist for interviews and focus groups. Int J Qual Healthc.

[36] Glaser BG, Strauss AL (1967). The discovery of grounded theory: strategies for qualitative research.

[37] Fong W, Kwan YH, Yoon S, Phang J, Thumboo J, Leung Y, Ng S. Assessment of medical professionalism: preliminary results of a qualitative study. BMC Med Educ. 2020;20:27. 10.1186/s12909-020-1943-x PMC6993492 32000755

[38] ETHICAL CODE AND ETHICAL GUIDELINES FOR TCM PRACTITIONERS [https://www.moh.gov.sg/docs/librariesprovider4/guidelines/ethical-code-and-ethical-guidelines-for-tcmp_e_c.pdf].

[39] Quill TE, Brody H (1996). Physician recommendations and patient autonomy: finding a balance between physician power and patient choice. Ann Intern Med.

[40] Wang W-j, Zhang T (2017). Integration of traditional Chinese medicine and Western medicine in the era of precision medicine. J Integrative Med.

[41] Cheah J, Heng BH (2001). Implementing chronic disease management in the public healthcare sector in Singapore: the role of hospitals. World Hosp Health Serv.

[42] Chung VC, Yip BH, Griffiths SM, Yu EL, Liu S, Ho RS, Wu X, Leung AW, Sit RW, Wu JC, Wong SY (2015). Patients' experience of Chinese medicine primary care services: implications on improving coordination and continuity of care. Sci Rep.

[43] Fung FY, Linn YC (2015). Developing traditional Chinese medicine in the era of evidence-based medicine: current evidences and challenges. Evid Based Complement Alternat Med.

[44] Luo H, Li H, Wang Y, Yao S, Xu W (2018). Clinical practice guidelines for treating headache with traditional Chinese medicine: quality assessment with the appraisal of guidelines for research and evaluation II instrument. J Tradit Chin Med.

[45] Liu M, Zhang C, Zha Q, Yang W, Yuwen Y, Zhong L, Bian Z, Han X, Lu A (2017). A national survey of Chinese medicine doctors and clinical practice guidelines in China. BMC Complement Altern Med.

[46] Reisman D. Payment for health in Singapore. Singapore. Int J Soc Econ. 2006;33:132–59. https://www.emerald.com/insight/content/doi/10.1108/03068290610642229/full/html.

[47] Hilton SR, Slotnick HB (2005). Proto-professionalism: how professionalisation occurs across the continuum of medical education. Med Educ.

