# Supplementary material for: Professionalism in traditional Chinese medicine (TCM) practitioners: a qualitative study
Source: BMC Complement Med Ther. 2020 Nov 9;20:335. doi: 10.1186/s12906-020-03127-8 (PMC7653838; doi:10.1186/s12906-020-03127-8)
Supplement: Supplementary file 1 — Additional file 1. [file 12906_2020_3127_MOESM1_ESM.docx]

**In-depth Interview Topic Guide**

**INTRODUCTION (5-10 MINS)**

Good morning, we are trying to find out about medical professionalism. So, we will be asking some questions. The interview will last approximately 1.5 hours.

**INTERVIEW (60 MINS)**

1. How would you describe a “professional” physician in terms of specific behaviours and practices in Singapore?

Probe:

1. [if needed, explain] A professional physician can be defined by multiple characteristics of behaviours, and would probably mean more than a “good” physician. Today, our focus is on a characteristic of behaviours not simply of the individual. In your opinion, what would be the “elements” that define a “professional” physician in Singapore?
2. [if any of these dimensions are not covered spontaneously, ask about]

Note: Do not give specific examples.

Physician-patient relationship

Inter-professional relationship

Time management

Quality of care

Communication

Presentation and attire

System-based practice (related to working in various health care delivery settings: a resident must demonstrate the ability to coordinate patient care within the health care system relevant to their clinical specialty)

Knowledge and competency

c. Have you ever encountered a physician who displayed a “professional” behaviour? If so, tell me the details.

d. Have you ever encountered a physician who demonstrated “lapses in professional conduct”? If so, tell me the details.

2. Now we are changing the focus a little bit. Think about what we have talked on “what are the behaviors of professional physicians”; now we exploring “how” these qualities of physicians can be assessed. Here is a set of behaviors that we could possible assess the professional behaviors. What do you think of each item for assessing this concept of “medical professionalism”. Please think along each item in terms of whether they are relevant, and feasible in the Singapore’s cultural and health care context.

|  | **Physician-patient relationship skills** | **Clarification** |
| --- | --- | --- |
| 1 | Listened actively to patient | Listened carefully to patient including asking questions to clarify what the patient really meant |
| 2 | Showed interest in patient as a person | Treated the patient as a person and not just a patient, i.e. as a person with his/her own opinion, interests, priorities, and family concerns. |
| 3 | Recognized and met patient needs | Understood what the patient really needed and met that need. |
| 4 | Extended his/herself to meet patient needs | Made the extra effort to help the patient obtain what he/she needed |
| 5 | Ensured continuity of patient care | Ensured proper hand-over of care of patient, e.g. at handover rounds in the evening or when discharging the patient to the care of another physician |
| 6 | Advocated on behalf of a patient | Spoke up in support of the patient - e.g. made an appeal on behalf of the patient to get subsidy for medical care. |
| 11 | Maintained appropriate boundaries | Aware of and behaved appropriately according to social, religious and cultural norms in our multi-ethnic and multi-religious country. e.g. neither overly friendly or unfriendly to patients and colleagues. |
|  | **Reflective skills** |  |
| 7 | Demonstrated awareness of limitations | Recognized limitations of own knowledge and skills and asked for help. |
| 8 | Admitted errors/omissions | Owned up to mistakes made, including medical errors |
| 9 | Solicited feedback | Actively asked for feedback on his/her actions or management of patients |
| 10 | Accepted feedback | Open to criticisms and comments and had the humility to learn from feedback |
| 12 | Maintained composure in a difficult situation | Remained calm and rational during medical emergencies or when faced with difficult patients, relatives or colleagues. |
|  | **Time management** |  |
| 14 | Was on time | Was punctual and started clinic, ward round and other duties on time |
| 15 | Completed tasks in a reliable fashion | Met datelines and completed assigned work on time |
| 17 | Was available to colleagues | Extended help to colleagues when needed |
|  | **Interprofessional relationship skills** |  |
| 13 | Maintained appropriate appearance | Appeared clean, neat, groomed and dressed modestly |
| 16 | Addressed own gaps in knowledge and skills | Kept up to date in medical knowledge and skills. e.g. by reading journals, attending courses and conferences |
| 18 | Demonstrated respect for colleagues | Listened to their professional opinion and did not oppose colleagues in public. |
| 19 | Avoided derogatory language | Did not ridicule, insult or use foul language when talking about patients or colleagues. |
| 20 | Maintained patient confidentiality | Did not reveal confidential medical or private information about patients to anyone except to those directly involved with patient care. |
| 21 | Used health resources appropriately | Use of health resources judiciously, based on scientific evidence and with the understanding that health care resources are limited. |

Probe: [Show a card and read each item. For each item, use the following questions in a flexible manner]

1. Why did you think that [each item] is (or is not) important in assessing a “professional” physician?
2. What thoughts came to mind when reading this item?
3. Can you repeat this phrase in your own words?
4. What does it mean to be [e.g. advocated on behalf of a patient] to you?
5. Is this item feasible to be used in assessing a “professional” physician?

3. Is there anything else that we didn’t list here but you think is very important for the assessment of a “professional” physician in Singapore?

4. Out of these 21 items, which one do you feel is less important in assessing professionalism? Please choose 5 items.

Probe: [Show a paper with 21 items]

5. Is there anything else that you’d like to add to what you’ve said?

**Collection of Participant demographics data/ heatlh questionnaire (5 MINs)**

Could you kindly help us to fill in this page regarding some information about you? This may help us in analyzing the information you give today.

**Thank you all very much for your participation in our research.**
