# Supplementary material for: Professionalism in traditional Chinese medicine (TCM) practitioners: a qualitative study
Source: BMC Complement Med Ther. 2020 Nov 9;20:335. doi: 10.1186/s12906-020-03127-8 (PMC7653838; doi:10.1186/s12906-020-03127-8)
Supplement: Supplementary file 2 — Additional file 2. [file 12906_2020_3127_MOESM2_ESM.docx]

**Supplementary Table 1.** Detailed demographic profile of TCM practitioners who participated in the in-depth interviews (n=27)

| Subject ID | Age (years) | Gender | Duration of practice (years) | Nature of place of practice  (For-profit/Voluntary) |
| --- | --- | --- | --- | --- |
| T01 | 32 | Female | 7 | Voluntary |
| T02 | 32 | Female | 7 | For-profit |
| T03 | 31 | Female | 7 | Voluntary |
| T04 | 31 | Male | 5 | Voluntary |
| T05 | 31 | Male | 5 | Voluntary |
| T06 | 33 | Female | 8 | For-profit |
| T07 | 31 | Male | 7 | For-profit |
| T08 | 49 | Female | 25 | For-profit |
| T09 | 40 | Female | 6 | Voluntary |
| T10 | 33 | Female | 7 | Voluntary |
| T11 | 61 | Female | 12 | Voluntary |
| T12 | 31 | Female | 7 | Voluntary |
| T13 | 27 | Female | 3 | Voluntary |
| T14 | 33 | Female | 9 | For-profit |
| T15 | 33 | Male | 7 | Voluntary |
| T16 | 66 | Female | 44 | For-profit |
| T17 | 57 | Female | 28 | For-profit |
| T18 | 33 | Male | 7 | Voluntary |
| T19 | 64 | Male | 37 | Voluntary |
| T20 | 27 | Female | 2 | For-profit |
| T21 | 68 | Male | 41 | For-profit |
| T22 | 67 | Female | 42 | Voluntary |
| T23 | 48 | Female | 13 | For-profit |
| T24 | 46 | Male | 14 | For-profit |
| T25 | 77 | Male | 40 | For-profit |
| T26 | 69 | Male | 14 | For-profit |
| T27 | 57 | Male | 29 | For-profit |
